# Effect of Boiling and Roasting Treatments on the Nutrients, Lipid Quality, and Flavor of Peanuts

**DOI:** 10.1002/fsn3.4509

**Published:** 2024-10-03

**Authors:** Ying Zhang, Weichao Zhu, Yuanbao Jin, Jiyuan Xu, Wenlong Zhou, Tingting Shen, Anshu Yang, Zhihua Wu, Hongbing Chen

**Affiliations:** ^1^ State Key Laboratory of Food Science and Resources Nanchang University Nanchang China; ^2^ College of Food Science and Technology Nanchang University Nanchang China; ^3^ Modern Agriculture and Forestry Engineering College Ji'an Vocational and Technical College Ji'an Jinagxi China; ^4^ School of Food and Biological Engineering Jiangsu University Zhenjiang Jiangsu China; ^5^ Sino‐German Joint Research Institute Nanchang University Nanchang China

**Keywords:** boiling, flavor, nutrients, peanut, roasting

## Abstract

Roasted peanuts (OPs) are more often used in daily life than boiled peanuts (BPs); the main reason may be related to the pleasurable flavor in OPs. This study comparatively investigated the effects of boiling and roasting on nutritional components contents, taste, and aroma to reveal the advantages of roasting in terms of the nutrition and flavor of peanuts. Results showed that boiling increased fat content to 54.47% of peanuts, diluted or reduced sugar content to an undetectable level, noticeably changed the contents of poly/monounsaturated fatty acids, and significantly decreased the content of free amino acid (FAA, 1.05 mg/g peanuts) in peanuts. Roasting simply decreased the contents of sugar and FAA to 2.78 and 1.82 mg/g peanuts, respectively. Compared with roasting, boiling had more discernible effects on fatty acids and FAAs. Both BPs and OPs have good dietary lipid quality. In terms of flavor, OPs had more FAAs (2 types) that contributed remarkably to the taste of peanuts than BPs (0 types), more volatile substances (40 types) than BPs (12 types), and a higher number of key volatile aroma components (8 types) than BPs (3 types). That is, roasting induced a relatively small effect on nutritional components and gave peanuts a richer taste and stronger aroma than boiling; therefore, roasting is an ideal peanut processing method. This study proved the different changes in peanuts caused by boiling and roasting on a material basis and corroborated the irreplaceability of roasting in peanut processing.

## Graphical abstract

In this study, we investigated the changes in nutrients, lipid quality, and flavor of peanuts after boiling or roasting. Results showed that compared with boiling, roasting had lesser effects on nutritional components and remarkably improved the taste and aroma of peanuts. Therefore, roasting is an ideal processing for peanuts.

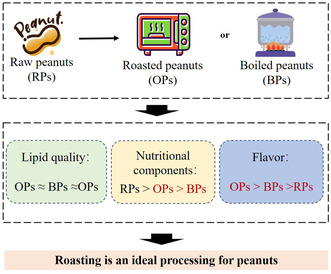

## 1 Introduction

Peanuts, whose annual yields are increasing worldwide (Fletcher and Shi 2016), are rich in lipid, protein, and carbohydrate (Syed et al. 2021). They have an oil content of approximately 50% and unsaturated fatty acid (UFA) content of 75%–82% (Zambiazi et al. 2007), with oleic acid and linoleic acids being the most abundant fatty acids (Chamberlin et al. 2014). Peanuts are also a high‐protein food, exhibiting a protein content of approximately 30%, and contain eight amino acids that are essential for the human body. Carbohydrate in peanut kernels accounts for approximately 20%, is mainly composed of starch, and includes sucrose, fructose, and glucose (Toomer 2018). Peanuts have a high edible value and are favored by food processors because of their high nutritional value.

Peanuts are also one of the eight major allergenic foods worldwide and can induce severe anaphylaxis. Boiling and roasting are common and simple processing methods for peanuts. Extreme boiling can reduce the allergenicity of peanuts (Tao et al. 2016). Roasting has a nonuniform effect on the allergenicity of peanuts (Wang et al. 2023; Zhang et al. 2019, and Zhang, Zhao, Su and Lin 2019) that does not affect the popularity of roasting in the preparation of peanut products. In the United States, 57% and 23% of peanuts are used for the preparation of peanut butter and roasted peanuts (OPs), respectively, and 19% of peanuts are used for desserts and ingredients (Qiang 2018). Considering that roasting is involved in the preparation of peanut butter, OPs, and other ingredients, more than 80% of peanuts can be assumed to be used for roasting. It is tempting to speculate that the main reason for the widespread adoption of roasting in peanut processing may be related to the pleasurable flavor of OPs. Up to now, data on the flavor of boiled peanuts (BPs) in previous reports are scarce. The flavor of OPs and BPs can be fully judged only by sensory evaluation by human subjects. Hence, discovering the flavor quality of BPs can increase knowledge of the chemistry of BP flavor and, more importantly, is crucial for proving the theoretical foundation for the widespread use of roasting in peanut processing.

Headspace solid‐phase microextraction (HS–SPME) is a volatile extraction technology that is superior to traditional extraction methods. Given its advantages of short operation time, small sample dosage, fast and flexible operation, good reproducibility, and high precision, it has become a popular sample pretreatment technology since its inception (Antignac et al. 2004). Gas chromatography–mass spectrometry (GC–MS) is commonly used for the analysis of volatile components in food due to its good limits of quantification, linearity, selectivity, matrix effects, recovery, and repeatability (Lopez et al. 2015). HS–SPME–GC–MS has been used for the extraction, separation, and identification of volatile compounds in various foods, such as tea and meat (Domínguez et al. 2019; Xu et al. 2023).

In this study, we comparatively analyzed the nutrients and flavors of BPs and OPs for the first time to increase knowledge of the chemistry of BP flavor, illustrate the advantages of roasting in terms of the nutrition and flavor of peanuts, and provide a theoretical basis for the widespread utilization of peanut roasting.

In this work, peanuts subjected to extended boiling were selected as a subject considering their reduced allergenicity. A previous study proved that an excessively low (149°C) or high (191°C and 204°C) roasting temperature negatively affected the overall flavor of OPs and increased ashy and total off notes (Shi et al. 2017). Therefore, peanuts roasted at a moderate temperature (170°C) with an ideal L* color value of 58–59 were selected. Protein, fat, and sugar contents were determined to evaluate the changes in peanut basic nutrients, and the fatty acid contents were detected to analyze the lipid quality after processing. The effect of free amino acids (FAAs) on the overall taste of peanuts was evaluated on the basis of taste activity value (TAV). Volatile flavor substances were detected by HS–SPME–GC–MS, and the main actors were analyzed in terms of relative odor activity value (ROAV). Finally, the effects of different thermal processing techniques on the taste and flavor of peanuts were analyzed by using cluster heatmaps to explore the different effects of thermal processing on peanut quality.

## 2 Materials and Methods

### 2.1 Sample Preparation

Peanuts (variety: ‘Huayu16’) were purchased from farmers in China. Dried peanuts were shelled, and peanut kernels that were undamaged, had good color, and consistent size were used as raw peanuts (RPs). In boiling processing, RP kernels were placed in boiling water and cooked for 210 min until they became soft. In roasting processing, RP kernels were placed in a roasting pan and roasted at 170°C for 10 min to obtain peanuts with an L* value of approximately 58, which meets the ideal color (Shi et al. 2017).

### 2.2 Determination of Total Protein, Fat, and Sugar Contents of Peanuts

#### 2.2.1 Determination of Total Protein Content

Total protein content was determined by using the Kjeldahl nitrogen method (Zhang et al. 2019). In detail, peanut powder was first digested with sulfuric acid in a digestion tube at 420°C for 1 h. After being cooled to room temperature, the digested solution was placed in an automated Kjeldahl analyzer (K9680; Hanon, China) to determine Kjeldahl nitrogen content. During distillation, ammonium vapor was entrapped in boric acid containing indicators, and the condensate was titrated with 0.1 M hydrochloric acid (HCl; XiLONG Scientific, China) until a light violet color was observed (Zhu et al. 2021 and Zhu, Niu and Xiao 2021). The volume of HCl was used to calculate the nitrogen content in the sample. The factor for the conversion of nitrogen content into protein content was 5.46.

#### 2.2.2 Determination of Fat Content

Fat content was detected through acid hydrolyzation. Peanut samples were hydrolyzed with HCl. Fat was then extracted by using ethanol and anhydrous ether (XiLONG Scientific, China), and anhydrous ether was recovered. The solvent in the receiving bottle was evaporated in a water bath until only 1–2 mL remained. The residual solvent was dried at 100°C ± 5°C for 1 h until it reached a constant weight (Guo et al. 2020).

#### 2.2.3 Determination of Sugar Content

Sugars in the samples were detected in accordance with the method of the National Standard of China (GB 5009.7–2016) and a previous study (Sun et al. 2019).

In brief, 5 g of the sample was weighed and crushed in a 100 mL plugged centrifuge tube, added with 50 mL of petroleum ether (XiLONG Scientific, China), mixed, deflated, shaken for 2 min, and centrifuged at 1800 rpm for 15 min. Petroleum ether was removed, and the abovementioned steps were repeated until most of the fat was removed. The residual petroleum ether was evaporated. The remaining samples were mixed with zinc acetate and potassium ferrocyanide solutions (Sigma‐Aldrich, Germany) for 30 min in an ultrasonic bath and filtered with a 0.45 μm microporous membrane. The filtrate was collected for HPLC analysis (LC‐20A; Shimadzu, Japan).

The chromatographic conditions were as follows: mobile phase: acetonitrile: water with a volume ratio of 70:30; mobile phase flow rate of 1.0 mL/min; column temperature of 40°C; and injection volume of 20 μL. The differential refractive index detector conditions included a temperature of 40°C. The evaporative light scattering detector conditions included a drift tube temperature of 80°C–90°C and nitrogen pressure of 350 kPa with the impactor in an off state. Quantification was performed with a calibration curve plotted with fructose, sucrose, maltose, lactose, and glucose (J&K Scientific, China), and the summation of these sugars was calculated.

### 2.3 Determination of the Fatty Acid and FAA Contents of Peanuts

#### 2.3.1 Analysis of Fatty Acids

The contents of fatty acids in samples were detected in accordance with the method of the National Standard of China (GB 5009.168–2016). Peanut samples were placed in a dry spiral glass tube, added with 10 mL of hydrochloric acid, and hydrolyzed for 40 min at 75°C. Fat was extracted from the samples by using hydrolysis–ether solution and saponified and methyl‐esterified under alkaline conditions to generate fatty acid methyl esters (Zhang et al. 2022). The percentage of fatty acid content was quantitatively determined by area normalization with a capillary column gas chromatograph (GC‐2010Plus; Shimadzu, Japan).

#### 2.3.2 Analysis of Amino Acid Profiles

A total of 200 mg of each peanut sample was hydrolyzed with 10 mL of 0.01 mol/L hydrochloric acid for 30 min. After filtration, 2 mL of the supernatant was mixed with 2 mL of 8% sulfosalicylic acid (Sigma‐Aldrich, Germany), allowed to stand for 15 min, and centrifuged at 3000 rpm for 20 min. The supernatant obtained after centrifugation was filtered through a 0.22 μm filter membrane and analyzed by using an automatic amino acid analyzer S‐433(D) (Sykam, Germany).

### 2.4 Analysis of the TAV of FAAs in Peanuts

The TAV of FAAs is used to identify amino acids that contribute considerably to the taste of peanuts (Fan et al. 2022) and is calculated by using Equation (1):

(1) $$\mathrm{TAV}=\frac{C_{1}}{C_{2}}$$

where *C*_*1*_ is the concentration of taste compounds (g/kg), and *C*_*2*_ is the taste threshold concentration (g/kg). When TAV > 1, the substance is considered to have an important modifying effect on the taste of the sample, and when TAV < 1, the substance is considered to have little contribution to the taste.

### 2.5 Identification of Volatile Flavor Compounds in Peanuts

The volatile flavor compounds in samples were detected via HS–SPME–GC–MS (Wang et al. 2019). A first‐use SPME DVB/CAR/PDMS‐coated SPME fiber (2 cm, Merck, Germany) was aged to no peak at the inlet of a GC system at 270°C for 40 min.

Peanuts were ground, weighed (2.0 g) into a headspace bottle, and equilibrated at 80°C for 30 min. Subsequently, the SPME fiber was inserted into the balanced sample vial for 40 min and pulled out. After the extraction was completed, the SPME fiber was inserted directly into the instrument inlet and desorbed at 250°C for 5 min in the splitless injection port of the GC system for analysis.

An Agilent 7890B–7000D model GC–MS system (Agilent, USA) with a fused silica capillary column (DB‐35MS 30 mm × 0.25 mm × 0.25 μm; Agilent, USA) was used for GC. The column temperature was set as follows: the initial temperature was 50°C, which was retained for 2 min; raised to 100°C at the rate of 3°C/min and held for 0 min; raised to 150°C at the rate of 20°C/min and held for 0 min; and finally raised to 250°C at the rate of 15°C/min and held for 5 min. The MS conditions were as follows: electronic impact ion source; ionization energy of 70 eV; ion source temperature of 230°C; quadrupole temperature of 150°C; and mass scanning range of 35–500 m/z. Data retrieval was conducted by using the National Institute of Standards and Technology library as the retrieval library. The relative content of each compound was calculated by using the area normalization method.

### 2.6 Analysis of the ROAV of Aroma Compounds in Peanuts

The threshold of each aroma compound was checked, and the ROAV of each aroma compound was calculated with Equation (2) on the basis of relative quantification (Xu et al. 2023), as follows:

(2) $$\mathrm{ROA}V_{i}\approx \frac{C_{i}}{C_{\max}}\times \frac{T_{\max}}{T_{i}}\times 100$$

where *C*_*i*_ is the relative content of aromatic compounds in peanuts (%); *T*_*i*_ is the aroma threshold of compounds (μg/kg), and *C*_max_ and *T*_max_ represent the relative contents of compounds with the greatest contribution to the overall flavor of the sample. All compounds have ROAV ≤ 100, and a high ROAV is indicative of the high contribution of a component to the overall flavor of a sample. Compounds with ROAV ≥ 1 were identified as key flavor compounds in samples.

### 2.7 Statistical Analysis

GraphPad Prism 8.0 software was used to process the data of repeated measurements, and variance analysis was performed to verify the significance of sample differences. Differences were considered significant when *p* values were less than 0.05. Clustering heatmaps were normalized by row by using Origin 2023 software.

## 3 Results and Discussion

### 3.1 Analysis of the Main Nutrients in Processed Peanuts

**FIGURE 1:**
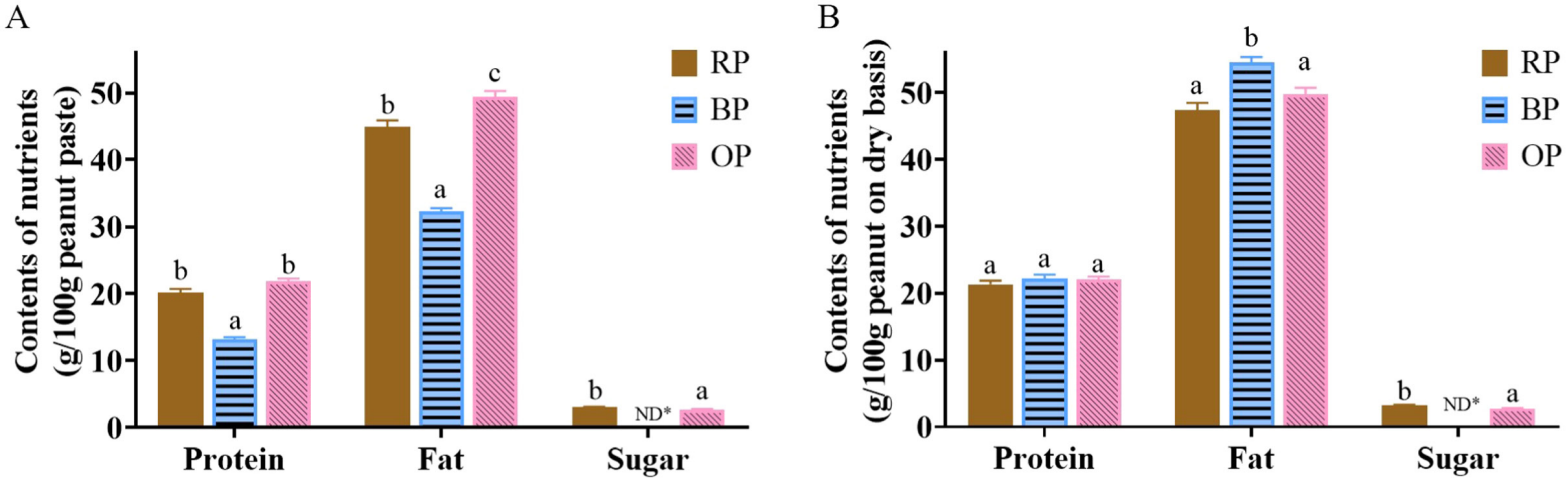
Contents of protein, fat, and sugar on wet (A) and dry basis (B) of three peanut samples. RP, raw peanut; BP, boiled peanut; OP, roasted peanut. The different lowercases indicated the significant difference (*p* values < 0.05) among samples. ND, not detectable.

Figure 1 shows the protein, fat, and sugar contents of peanut samples. The contents of protein, fat, and sugar in RPs on a wet basis were 20, 45, and 3 g/100 g peanuts, respectively, and were consistent with the previously reported values (Cong et al. 2007; Kuang et al. 2017). After boiling, the contents of protein and fat in equivalent weights of peanuts significantly decreased and reached 13.17% and 32.25%, respectively, which were also found in a previous study (Kemmler et al. 2024). The sugar content in peanuts was too low to be detected (< 0.2 g/100 g) due to loss to cooking water, the dilution effect caused by the increase in water, or material transformation. The protein content (21.84 g/100 g) of OPs was similar to that of RPs. The fat content of OPs was significantly higher (49.34 g/100 g) than that of RPs. The sugar content (2.75 g/100 g) of OPs was lower than that of RPs.

In terms of the moisture contents of peanut products, BPs showed a higher water content (40.78%) than RPs (5.29%), and OPs had the lowest moisture content (0.93%) among samples. In order to exclude the effect caused by moisture content, the contents of nutrients in peanuts on a dry basis, except for the undetected data for sugar in BPs, were calculated and are shown in Figure 1B. The results showed that compared with that of RPs, the protein content of BPs did not change. This finding was inconsistent with the data obtained on a wet basis and indicated that the effect of boiling treatment on peanut nutrients was related to the change in moisture content. Meanwhile, the fat content of BPs increased. A previous study (De Santiago et al. 2018) reported that the boiling treatment of food may exert a softening effect due to damage to cell wall components and may thus increase the efficiency of fat extraction under the condition of acid hydrolyzation. The sugar content of OPs decreased compared with that of RPs, likely due to sugar caramelization or Maillard reactions during roasting (Tas and Gökmen 2017).

Boiling and roasting had little effect on the loss of nutrients and may simply affect sugar content.

### 3.2 Evaluation of the Lipid Quality of Processed Peanuts

The components of fatty acids in the three types of peanut products are listed in Table S1. The fatty acids that were abundant in peanuts included C18:1 (oleic acid), C18:2 (linoleic acid), and C16:0 (cetylic acid). After boiling, the relative contents of C22:0 (docosanoic acid) and C18:1 (oleic acid) decreased, whereas that of C18:2 (linoleic acid) increased. After roasting, the relative content of C22:0 decreased, whereas those of C16:0 and C16:1 (palmitoleic acid) increased. Boiling and roasting prompted different changes in the contents of different fatty acids.

**FIGURE 2:**
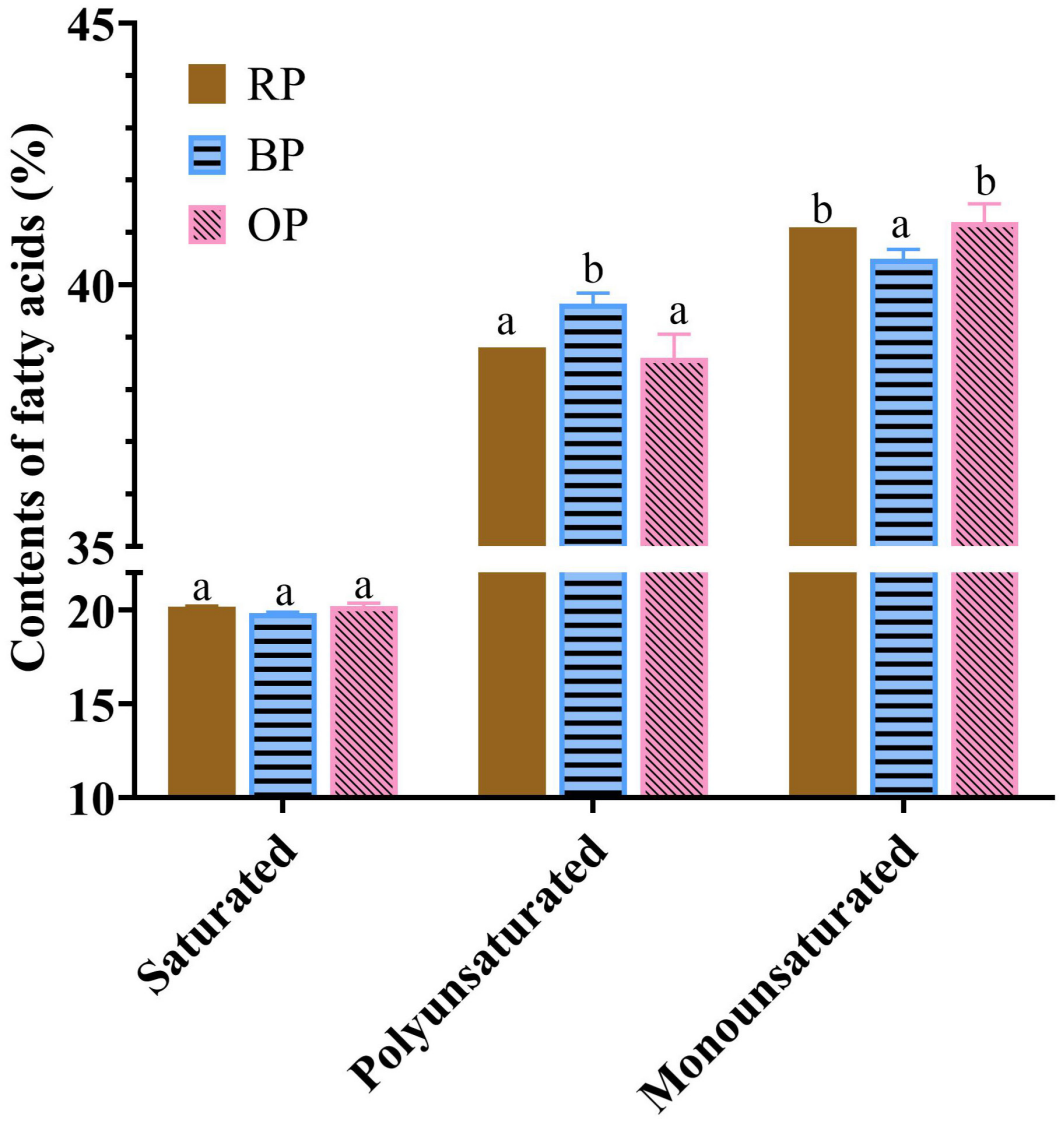
Composition of fatty acid of the three peanut samples. RP, raw peanut; BP, boiled peanut; OP, roasted peanut. The different lowercases indicated the significant difference (*p* values < 0.05) among samples.

In accordance with the structural characteristics of fatty acids, the contents of saturated, monounsaturated, and polyunsaturated fatty acids (PUFAs) were calculated to analyze the fatty acid composition of the three peanut samples. Figure 2 shows that UFAs accounted for approximately 80% of the total fatty acids in the three peanut samples. Notably, boiling treatment increased the content of PUFAs and decreased that of monounsaturated fatty acids (MUFAs), whereas roasting resulted in no remarkable change. These results disagreed with the findings obtained by a previous work (Guo et al. 2020), which found that boiling for 25 min decreased PUFA content and increased MUFA content, and that roasting at 160°C for 15 min decreased UFA content. This difference could be explained by differences in processing conditions and/or peanut variety. The ratio of PUFAs to saturated fatty acids (SFAs) is a relevant nutritional indicator (Rincón‐Cervera et al. 2019). In this study, all three samples showed favorable PUFA:SFA ratios (above the recommended minimum value of 0.45, Table S1), indicating good dietary lipid quality.

Compared with roasting, boiling had more discernible effects on fatty acids.

### 3.3 Evaluation of the FAAs of Processed Peanuts

**FIGURE 3:**
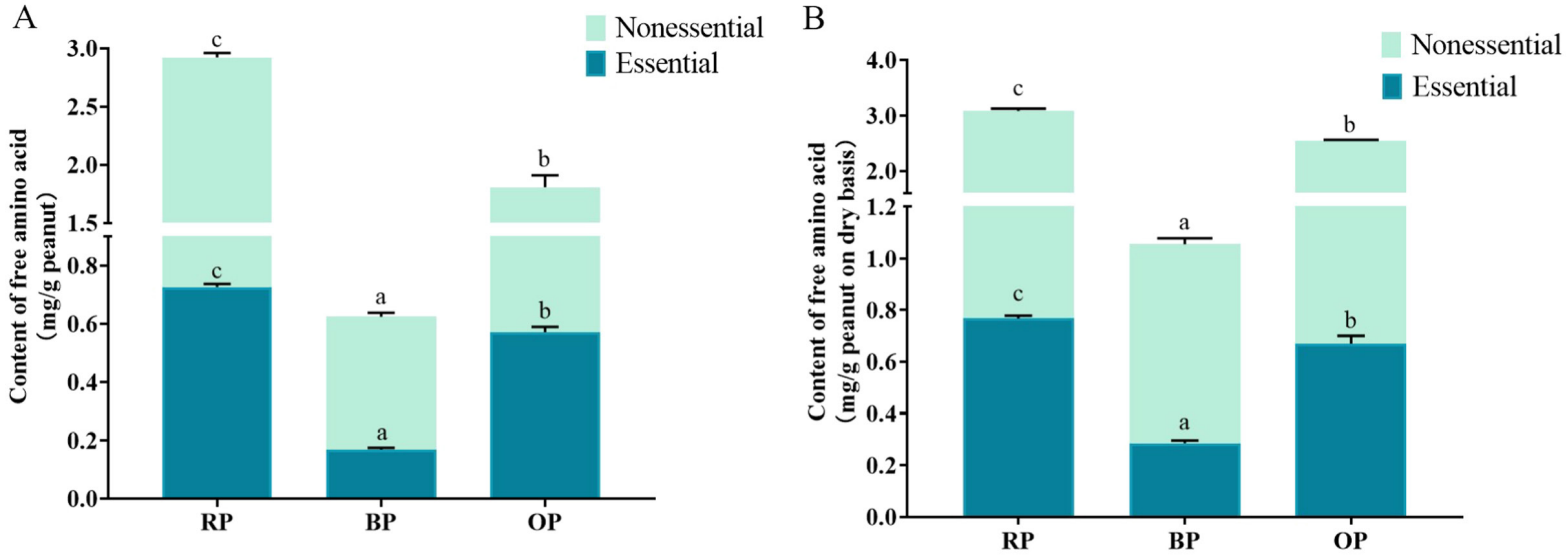
Contents nonessential and essential free amino acids on wet (A) and dry basis (B) of the three peanut samples. RP, raw peanut; BP, boiled peanut; OP, roasted peanut. The different lowercases indicated the significant difference (*p* values < 0.05) among samples.

The contents of 16 out of 20 protein FAAs were detected. The accumulated content of FAAs was considered as the content of total FAA (t‐FAA), which was nearly 3.0 mg/g peanuts in RPs. t‐FAA content, either on a wet or dry basis, decreased significantly after thermal processing (Figure 3) and especially after boiling. These results proved that thermal processing resulted in the large loss of FAAs (Guo et al. 2020).

**FIGURE 4:**
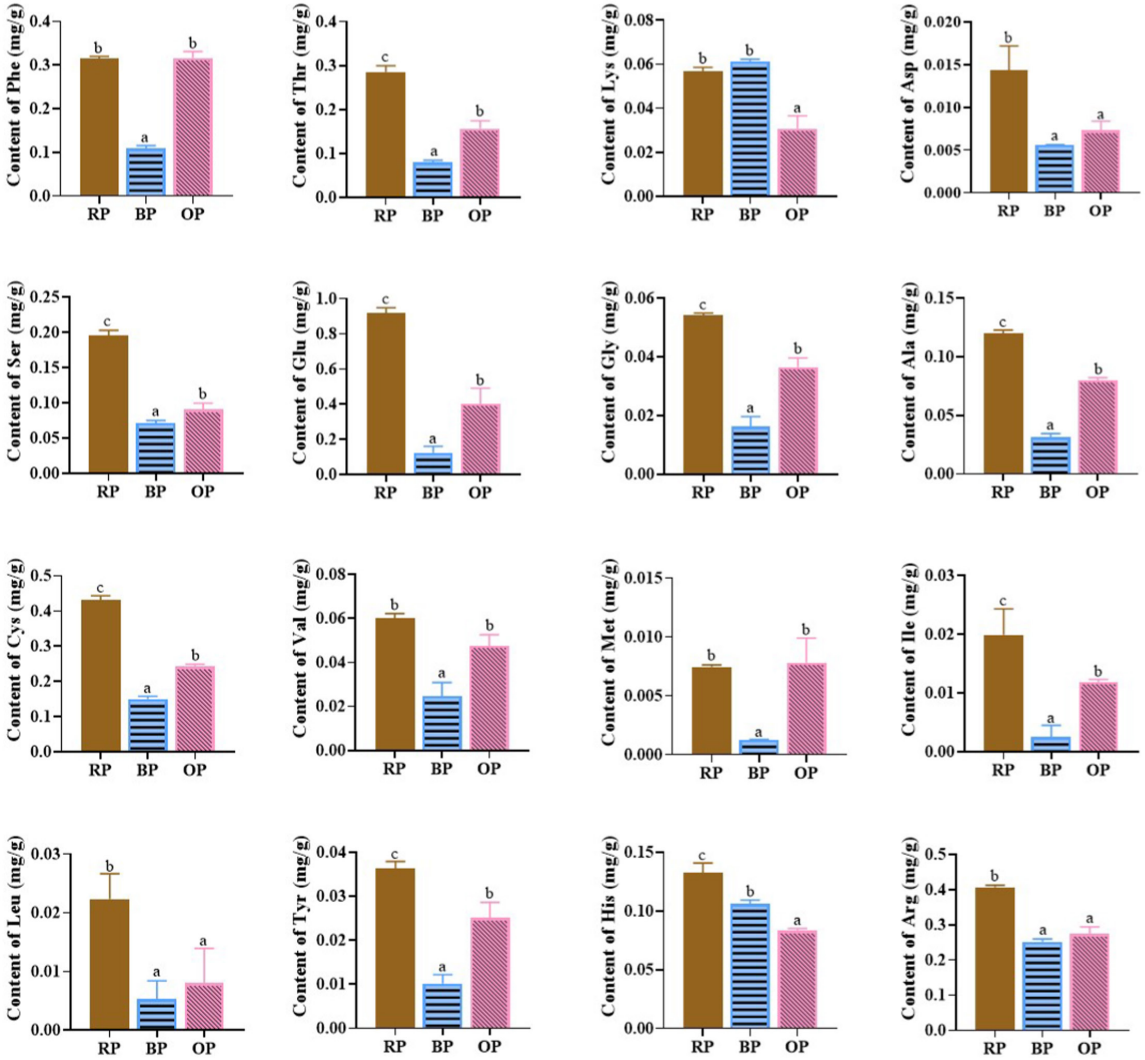
Contents of individual free amino acids in three peanut samples. RP, raw peanut; BP, boiled peanut; OP, roasted peanut. The different lowercases indicated the significant difference (*p* values < 0.05) among samples.

In terms of the contents of individual FAAs (Figure 4), different amino acids showed different levels of reduction under different processing conditions. Almost all FAAs, except for lysine, significantly decreased in BPs relative to in RPs. The depletion of FAAs in BPs may be due to loss to cooking water or aroma substance production.

FAAs, except for phenylalanine, methionine, and valine, were lower in OPs than in RPs. The decrease in FAAs in OPs may be related to the formation of aroma substances. The amino acids lysine and arginine have two susceptible free amino groups, which react with reducing sugars or α‐dicarbonyl compounds to form volatiles (Yu et al. 2021). Both FAAs significantly decreased in OPs. The decrease in glutamic acid and aspartic acid may also involve the precursors of important volatile components in peanuts that can react with 2‐deoxyglucose to produce pyrazines and phenol (Lu, Yu, and Ho 1997).

Compared with roasting, boiling led to the greater loss of FAAs in peanuts.

### 3.4 TAV Analysis of Taste Substances in Processed Peanuts

As shown in Table 1, the FAAs of peanuts can be divided into 2 types of umami FAAs, 4 types of sweet FAAs, and 10 types of bitter FAAs in accordance with taste. RPs had three amino acids with TAV > 1, namely, glutamic acid, which has an umami taste, and histidine and arginine, which have a bitter taste. Among these FAAs, glutamic acid showed the highest TAV. The TAVs of FAAs in BPs were low (< 1), indicating that amino acid components mainly played auxiliary roles in the taste of BPs. OPs had two amino acids, namely, glutamic acid and arginine, with TAV > 1. These amino acids exerted important modifying effects on the taste of peanuts. On the basis of TAVs, OPs presented a richer taste than BP.

**TABLE 1:** The TAV values of free amino acids in three peanut samples.

Taste	Free amino acid	Threshold (g/kg)	TAV		
			RPs	BPs	OPs
Umami	Asp	1.00	0.04	0.01	0.01
	Glu	0.30	6.63	0.63	2.73
Sweet	Thr	2.60	0.24	0.05	0.12
	Ser	1.50	0.28	0.07	0.12
	Gly	1.30	0.09	0.02	0.05
	Ala	0.60	0.43	0.08	0.27
Bitter	Cys	—	—	—	—
	Val	0.40	0.33	0.10	0.25
	Met	0.30	0.07	0.00	0.03
	Ile	0.90	0.04	0.00	0.02
	Leu	1.90	0.03	0.01	0.01
	Tyr	—	—	—	—
	Phe	0.90	0.76	0.19	0.71
	His	0.20	1.45	0.85	0.85
	Lys	0.50	0.24	0.20	0.12
	Arg	0.50	1.76	0.78	1.12

Abbreviations: BPs, boiled peanuts; OPs, roasted peanuts; RPs, raw peanuts.

### 3.5 Identification of Volatile Compounds in Processed Peanuts

**FIGURE 5:**
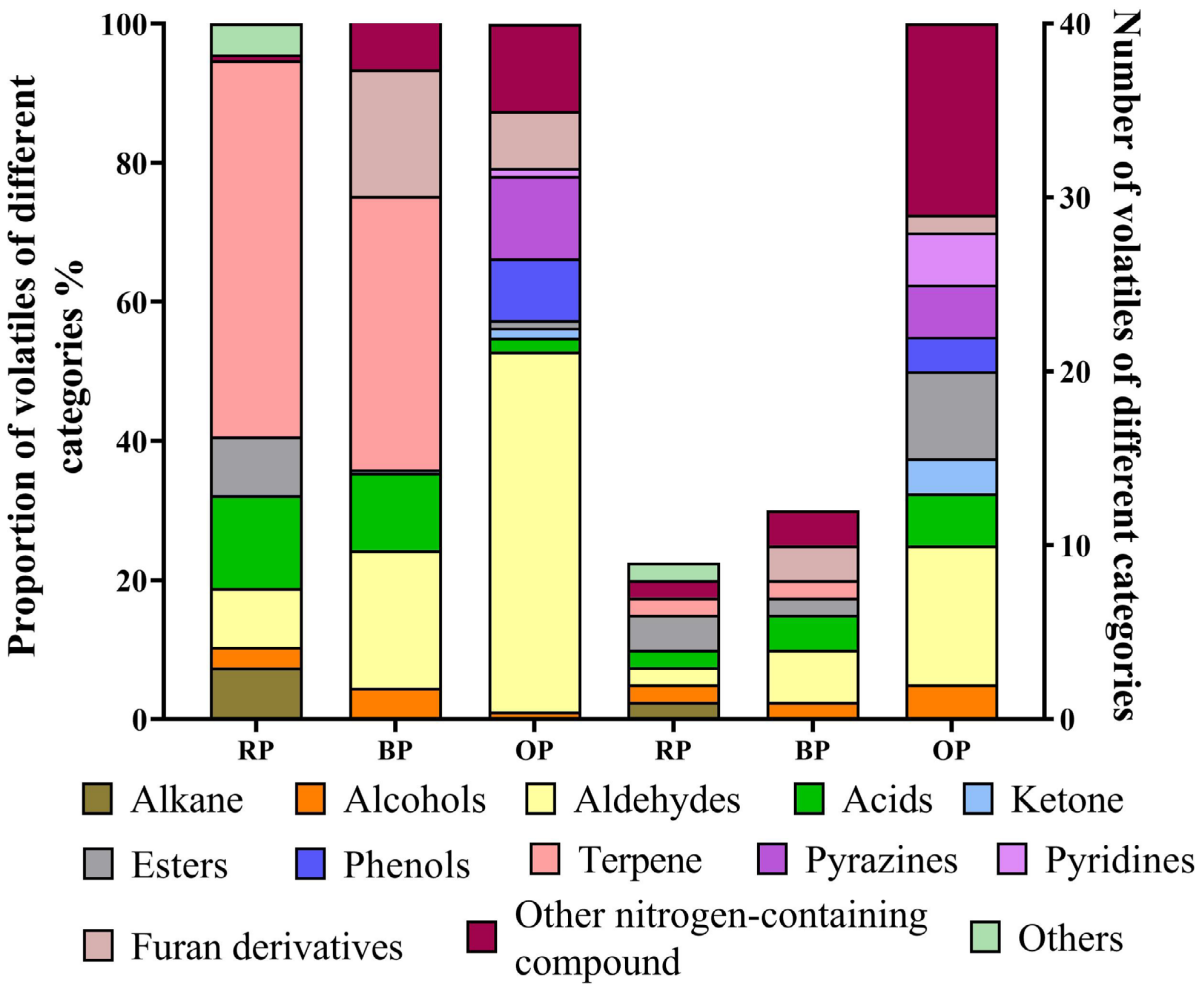
The proportion and number of volatiles of different categories identified by GC–MS in three peanuts.

A total of 49 volatile compounds in the three peanut samples were identified by using GC–MS (Table S2). The number and proportions of the volatile compounds are shown in Figure 5. All the identified volatiles belonged to 13 categories. The analysis of volatile substances in peanuts treated by using different processing methods showed that 9, 12, and 40 volatile substances were detected in RPs, BPs, and OPs, respectively. After processing, the number of volatile flavor substances in peanuts increased. Between the two thermal processing methods, roasting produced the greatest amount and variety of volatile components.

In RPs, terpene (54.07%) was the major volatile, followed by acids (13.39%), alkanes (7.45%), aldehydes (8.47%), and alcohols (2.92%). D‐limonene and oleic acid were the most abundant compounds in RPs, and D‐limonene was the only detected terpene.

In BPs, terpenes were also the major volatiles (39.29%), and only D‐limonene, which was lower in BPs than in RPs, was present. After boiling, the contents of alkanes and esters in peanuts reduced and that of acids decreased to 11.16%, whereas the number of acids increased to 2. In contrast to alkanes, terpenes, and esters, which significantly declined, other volatile components increased in BPs. The content of alcohols increased to 4.56% and that of aldehydes increased to 19.74%. Benzeneacetaldehyde, the major aldehyde in BPs, can be formed through the degranulation of phenylalanine via the Strecker pathway in the Maillard reaction (Zamora, Alcón, and Hidalgo 2013). Nonanal is thought to result from the thermal oxidation or degradation of oleic acids (Peng et al. 2023). Meanwhile, boiling promoted the formation of nitrogen‐containing compounds, including furan derivatives, such as 2,3‐dihydro‐benzofuran and 2‐pentyl‐furan. Furan derivatives could be mainly derived from the oxidation of lipids and Maillard reaction of precursor proteins and amino acids (Lu et al. 2022). 2‐Pentyl‐furan could be derived from the oxidation/degradation of lipids and is always detected in fruits (Kettlitz et al. 2019).

In OPs, aldehyde was the major volatile (51.75%) and benzeneacetaldehyde was the most abundant volatile component. After roasting, the number of aldehydes increased, and some ketones, which may be the secondary products of lipid oxidation (Barriuso, Astiasarán, and Ansorena 2013), were identified. The relative contents of esters and acids decreased, whereas the number of these two types of substances increased. This effect may indicate that numerous complex reactions occurring in OPs. Meanwhile, similar to that in BPs, 2,3‐dihydro‐benzofuran was detected in OPs. This compound had been detected in peanut oil (Hu et al. 2021) and was found at high levels in roasted large peanut seeds (Eker, Darici, and Cabaroglu 2023). In contrast to RPs and BPs, OPs contained phenols, pyridines, and pyrazines, indicating that further Maillard reactions occurred in OPs (Zhang et al. 2023). Pyrazines are important volatile Maillard reaction products that are mainly formed between α‐dicarbonyl compounds and amino compounds through Strecker degradation (Yu et al. 2021). The types of pyrazines related to the sources of amino groups, trimethylpyrazine is likely to be produced when the N‐terminal FAA of a peptide is glycine, alanine, or serine, whereas unsubstituted pyrazine is always produced in experiments with FAAs (Van Lancker, Adams, and De Kimpe 2012). Meanwhile, D‐limonene was not detected in OPs. Chunbo Guan et al. illustrated that D‐limonene first appeared and later disappeared with the prolongation of roasting time (Guan et al. 2022), suggesting that D‐limonene may be converted into other flavor substances under roasting conditions.

Although boiling and roasting induced the occurrence of the Maillard reaction, their products were different. Compared with BPs, OPs contained more aldehydes and nitrogen‐containing compounds; lower contents but a higher number of acids and alcohols; and additional phenols, ketones, and pyrazine volatiles. Roasting induced more complex reactions and volatiles in peanuts than boiling.

### 3.6 Analysis of the Key Aroma Compounds of Processed Peanuts

Although thousands of volatile compounds are found in food, only a few contribute substantially to food flavor. The volatile components that play a key role in the overall flavor of food are known as key aroma compounds (Zhu, Niu, and Xiao 2021). The ROAV of each volatile flavor component can be calculated in accordance with the relative content and threshold of the compound to evaluate its contribution to the flavor of food (Xu et al. 2023). The ROAVs of the identified volatile flavor components in this study are shown in Table 2. Given the difference in the contents and thresholds of volatile flavor substances in peanuts subjected to different processing methods, the key flavor substances that acted as the main flavor volatiles also differed.

**TABLE 2:** The ROAV values of the main volatile compounds in three peanut samples.

Volatile compounds	Odor description	Thresold (μg/kg)	ROAV		
			RP	BP	OP
D‐Limonene	Citrus, mint	210.00	100.00	100.00	nd
Benzeneacetaldehyde	Berry, geranium, honey, nut, pungent	350.00	9.40	22.71	100.00
Phenylethyl alcohol	Fruit, honey, lilac, rose, wine	122.00	9.30	nd	nd
Nonanal	Fat, floral, green, lemon	300.00	nd	6.02	nd
(*E*,*E*)‐2,4‐Nonadienal	Cereal, fat, wet wool	1.50	nd	nd	54.81
Methylpyrazine	Cocoa, hazelnut, popcorn, roasted	60.00	nd	nd	44.81
Furaneol	Burnt, caramel, cotton candy, honey	30.00	nd	nd	28.23
2‐Ethyl‐5‐methylpyrazine	Fruit, green	1000.00	nd	nd	4.92
Benzaldehyde	Bitter almond, burnt sugar, malt, roasted pepper	600.00	nd	nd	4.41
1‐(2‐pyridinyl)‐Ethanone	Popcorn, roasted nut	100.00	nd	nd	2.47
Trimethylpyrazine	Cocoa, earth, must, potato, roast	1000.00	nd	nd	2.11

*Note:* Odor description information can be found in https://www.femaflavor.org. Odor description can be found in the literature named Compilations of Odor Threshold Values in Air, Water and Other Media (Second Enlarged and Revised Edition).

Abbreviation: ND, not detectable.

RPs contained three key volatile substances (ROAV > 1), namely, D‐limonene (100), benzeneacetaldehyde (9.4), and phenylethyl alcohol (9.3). D‐limonene provides citrus and mint odors (Xiao et al. 2020). Benzeneacetaldehyde confers a nutty aroma to peanuts (Peng et al. 2023). Phenylethyl alcohol provides sweet and floral odors to peanuts (Garruti et al. 2006). These substances conferred RPs with a strong citrus odor and light nutty and floral odors.

Three key volatile substances (ROAV > 1) were found in BPs: D‐limonene (100), benzeneacetaldehyde (22.71), and nonanal (6.02). Similar to that in RPs, D‐limonene in BPs had the highest ROAV; it exhibits citrus, mint, and lemon‐like odors (Xiao et al. 2020). Benzeneacetaldehyde in BPs showed higher ROAVs than in RPs. Both aldehydes contributed a strong nutty aroma to peanuts. The ROAV results indicated that boiling enhanced the nutty aroma of peanuts.

OPs had 8 key volatile substances (ROAV > 1), with benzeneacetaldehyde, (*E*,*E*)‐2,4‐nonadienal, furaneol, and methylpyrazineare being the most important aroma components (ROAV > 28). In addition, 1‐(2‐pyridinyl)‐ethanone, 2,5‐dimethylpyrazine, 2‐ethyl‐5‐methylpyrazine, and benzaldehyde were the main aroma components in OPs (ROAV > 1). Benzeneacetaldehyde was the most abundant compound, together with (*E,E*)‐2,4‐nonadienal (cereal and fat aromas) and benzaldehyde (bitter almond, burnt sugar, and malt aromas), which conferred a strong nutty and caramel aroma to peanuts (Wang, Adhikari, and Hung 2017). Furthermore, pyrazines containing methylpyrazine, 2‐ethyl‐5‐methylpyrazine, and trimethylpyrazine contributed roasted and nutty aromas to peanuts (Spada et al. 2021). They are considered as key volatiles in OPs oil (Hu et al. 2021) and have also been found in roasted defatted tiger nut flour (Guan et al. 2022). These substances indicated that OPs had a caramel and roasted nutty aroma.

Comparing the two thermal processing methods revealed remarkable differences in aroma substances. The aroma substances with roasted nutty aromas produced by the Maillard reaction contributed to the unique aroma of OPs.

### 3.7 Comprehensive Analysis of the Nutritional Loss and Flavor of Processed Peanuts

**FIGURE 6:**
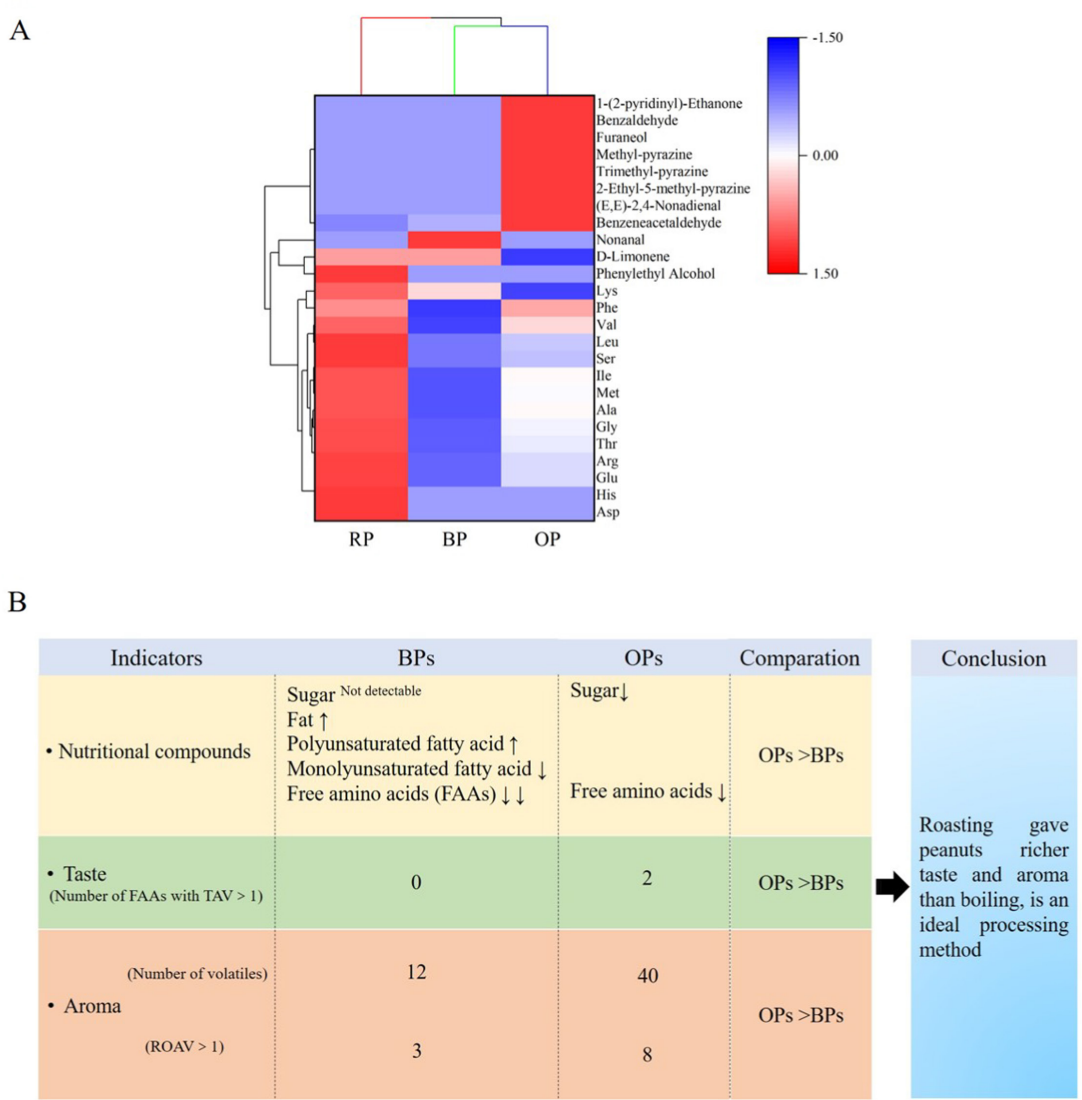
Cluster heatmap of changes in TAV and ROAV values of flavor substances in three peanuts (A). Blue color indicates low content, while red color indicates high content. Comprehensive schematic diagrams for effects of different treatments on nutritional compounds and flavor in peanuts (B).

A common merging algorithm for hierarchical clustering was used to determine the similarity between each category of data points and all data points. A small distance is indicative of high similarity. In Figure 6A, blue indicates low contents, whereas red indicates high contents in the three samples. Different colors in samples indicate differences in the flavors of samples. The differences between RPs and OPs were largest. Boiling exerted a negative effect on taste and did not improve the aroma of peanuts. The taste of OPs was between those of RPs and BPs. Moreover, among samples, OPs had the richest aroma. The comprehensive comparison of the effects of different treatments on the nutritional compounds and flavor of peanuts is shown in Figure 6B. Roasting induced a lesser loss of FAAs than boiling and did not result in remarkable changes in the content of PUFAs/MUFAs in peanuts. In terms of flavor, OPs had more FAAs with TAV > 1 and more volatiles with ROAV > 1 than BPs, indicating that OPs had a richer taste and aroma than BPs. Therefore, roasting is an ideal peanut processing method.

## 4 Conclusion

The nutrients and flavor profiles of peanuts subjected to different processes changed differently. Compared with boiling, roasting had lesser effects on fatty acids and FAAs and induced relatively small effects on nutritional components. Both OPs and BPs showed good dietary lipid quality. In terms of flavor, OPs had a richer taste and aroma than BPs, being strong roasted peanutty. Therefore, roasting is an ideal processing for peanuts.

## Author Contributions

**Ying Zhang:** conceptualization (equal), data curation (lead), formal analysis (equal), methodology (equal), software (lead), writing – original draft (lead), writing – review and editing (lead). **Weichao Zhu:** data curation (equal), software (equal). **Yuanbao Jin:** conceptualization (equal), funding acquisition (equal). **Jiyuan Xu:** data curation (equal), methodology (equal). **Wenlong Zhou:** data curation (equal), formal analysis (equal). **Tingting Shen:** data curation (equal), methodology (equal). **Anshu Yang:** data curation (equal), formal analysis (equal). **Zhihua Wu:** conceptualization (equal), data curation (equal), funding acquisition (lead), project administration (equal), resources (equal), supervision (lead), writing – original draft (equal), writing – review and editing (equal). **Hongbing Chen:** data curation (equal), project administration (equal).

## Conflicts of Interest

The authors declare no conflicts of interest.

## Supporting information


Table S1.
Table S2.

## Data Availability

The data that support the findings of this study are available from the corresponding author upon reasonable request.
