# Supplementary material for: Effect of Boiling and Roasting Treatments on the Nutrients, Lipid Quality, and Flavor of Peanuts
Source: Food Sci Nutr. 2024 Oct 3;12(11):9314–24. doi: 10.1002/fsn3.4509 (PMC11606846; doi:10.1002/fsn3.4509)
Supplement: Supplementary file 1 — Table S1. Table S2. [file FSN3-12-9314-s001.docx]

**Supplementary material**

TABLE S1 The relative contents of fatty acids in three peanut samples.

| Fatty acid | Relative content (%) | | |
| --- | --- | --- | --- |
|  | RPs | BPs | OPs |
| C12: 0 | ND | ND | 0.05±0.001 |
| C22: 1n9 | 0.03±0.000 | 0.03±0.001 | 0.05±0.002 |
| C18:3n3 | 0.04±0.000 | 0.04±0.001 | 0.04±0.001 |
| **C16:1** | **0.04±0.000^a^** | **0.04±0.000^a^** | **0.07±0.010^b^** |
| C17: 0 | 0.05±0.002 | 0.07±0.004 | 0.07±0.004 |
| C14: 0 | 0.06±0.001 | 0.06±0.002 | 0.10±0.003 |
| C20:1 | 0.60±0.010 | 0.49±0.010 | 0.53±0.020 |
| C24: 0 | 0.92±0.010 | 0.82±0.060 | 0.81±0.020 |
| C20: 0 | 1.35±0.010 | 1.25±0.010 | 1.22±0.060 |
| **C22: 0** | **1.95±0.010^b^** | **1.72±0.060^a^** | **1.66±0.060^a^** |
| C18:0 | 4.01±0.010 | 4.02±0.020 | 4.18±0.120 |
| **C16: 0** | **11.80±0.000^a^** | **11.90±0.100^ab^** | **12.17±0.150^b^** |
| **C18:1** | **40.40±0.000^b^** | **39.97±0.120^a^** | **40.53±0.400^b^** |
| C18: 1n9c | 40.40±0.000^b^ | 39.97±0.120^a^ | 40.53±0.400^b^ |
| **C18:2** | **38.74±0.060^a^** | **39.60±0.170^b^** | **38.53±0.500^a^** |
| C18: 2n6c | 38.74±0.060^a^ | 39.60±0.170^b^ | 38.53±0.500^a^ |
| C18:3 | 0.05±0.000 | 0.04±0.001 | 0.04±0.001 |
| Saturated fatty acid (SFA) | 20.16±0.060 | 19.83±0.060 | 20.21±0.170 |
| PUFA/SFA | 1.92±0.006 | 2.00±0.020 | 1.91±0.040 |

**^†^** RPs: raw peanuts, BPs: boiled peanuts, OPs: roasted peanuts. The different lowercases indicated the significant difference among samples.

TABLE S2 Volatile compounds identified in three peanut samples by GC-MS.

|  | Volatile Compounds | | RT | | CAS | | Relative contents | | | | | | |
| --- | --- | --- | --- | --- | --- | --- | --- | --- | --- | --- | --- | --- | --- |
|  |  |  |  |  |  |  | Raw | | Boil | | | Roast | |
| **Alkane** | | | | | | | | | | | | | |
| **1** | 7,7-Diethylheptadecane | | 22.77 | | 1000360-41-5 | | 7.45 | | **----** | | | **----** | |
| **Sum** |  | |  | |  | | **7.45** | | ---- | | | ---- | |
| **Terpene** | | | | | | | | | | | | | |
| **1** | D-Limonene | | 17.86 | | 5989-27-5 | | 54.07 | | 39.29 | | | **----** | |
| **Sum** |  | |  | |  | | **54.07** | | **39.29** | | | ---- | |
| **Alcohol** | | | | | | | | | | | | | |
| **1** | Phenylethyl Alcohol | | 22.53 | | 60-12-8 | | 2.92 | | **----** | | | **----** | |
| **2** | *Z*,*Z*-2,5-Pentadecadien-1-ol | | 7.74 | | 139185-79-8 | | **----** | | 4.56 | | | 0.38 | |
| **3** | 3-Furanmethanol | | 13.17 | | 4412-91-3 | | **----** | | **----** | | | 0.69 | |
| **Sum** |  | |  | |  | | **2.92** | | **4.56** | | | **1.07** | |
| **Aldehyde** | | | | | | | | | | | | | |
| **1** | Benzeneacetaldehyde | | 20.88 | | 122-78-1 | | 8.47 | | 14.87 | | | 42.57 | |
| **2** | Nonanal | | 21.03 | | 124-19-6 | | **----** | | 3.38 | | | **----** | |
| **3** | Undecanal, 2-methyl- | | 3.76 | | 110-41-8 | | **----** | | **----** | | | 0.33 | |
| **4** | 2,4-Heptadienal, 2,4-dimethyl- | | 19.52 | | 42452-48-2 | | **----** | | **----** | | | 0.50 | |
| **5** | Benzeneacetaldehyde,.alpha.-ethylidene- | | 24.41 | | 4411-89-6 | | **----** | | **----** | | | 1.21 | |
| **6** | (p-Hydroxyphenyl)glyoxal | | 25.51 | | 24645-80-5 | | **----** | | **----** | | | 3.06 | |
| **7** | Benzaldehyde | | 17.74 | | 100-52-7 | | **----** | | **----** | | | 3.22 | |
| **8** | 2,4-Nonadienal,(*E*,*E*)- | | 15.89 | | 5910-87-2 | | **----** | | **----** | | | 0.10 | |
| **9** | 3-Hydroxy-2-(2-methylcyclohex-1-enyl)propionaldehyde | | 22.15 | | 1000186-24-5 | | **----** | | **----** | | | 0.75 | |
| **10** | Tridecanedial | | 19.52 | | 63521-76-6 | | **----** | | 1.49 | | | **----** | |
| **Sum** |  | |  | |  | | **8.47** | | **19.74** | | | **51.75** | |
| **Ketone** | | | | | | | | | | | | | |
| **1** | 4H-Pyran-4-one, 2,3-dihydro-3,5-dihydroxy-6-methyl- | | 23.37 | | 28564-83-2 | | **----** | | **----** | | | 0.47 | |
| **2** | Furaneol | | 21.89 | | 3658-77-3 | | **----** | | **----** | | | 1.03 | |
| **Sum** |  | |  | |  | | **----** | | **----** | | | **1.50** | |
| **Acid** | | | | | | | | | | | | | |
| **1** | Oleic Acid | | 1.29 | | 112-80-1 | | 13.39 | | 10.46 | | | 1.16 | |
| **2** | Oxiraneoctanoic acid,3-octyl-, cis- | | 25.65 | | 24560-98-3 | | **----** | | **----** | | | 0.75 | |
| **3** | Dodecanoic acid, 3-hydroxy- | | 4.87 | | 1883-13-2 | | **----** | | **----** | | | 0.08 | |
| **4** | cis-7-Hexadecenoic acid | | 20.74 | | 2416-19-5 | | **----** | | 0.24 | | | **----** | |
| **Sum** |  | |  | |  | | **13.39** | | **10.70** | | | **1.99** | |
| **Ester** | | | | | | | | | | | | | |
| **1** | 7-Methyl-*Z*-tetradecen-1-ol acetate | | 21.81 | | 1000130-99-6 | | 5.84 | | **----** | | | 0.08 | |
| **2** | Undecanoic acidpropyl ester, 10-hydroxy-11-morpholin-4-yl- | | 24.09 | | 1000303-32-0 | | **----** | | **----** | | | 0.45 | |
| **3** | 10-Methyl-8-tetradecen-1-olacetate | | 20.18 | | 1000131-36-1 | | **----** | | **----** | | | 0.28 | |
| **4** | Octadecanoic acid,ethenyl ester | | 20.19 | | 111-63-7 | | **----** | | **----** | | | 0.17 | |
| **5** | Glycidyl (*Z*)-9-Heptadecenoate | | 22.30 | | 1000465-63-2 | | **----** | | 0.47 | | | **----** | |
| **6** | Dasycarpidan-1-methanol, acetate (ester) | | 24.13 | | 55724-48-6 | | 2.62 | | **----** | | | **----** | |
| **7** | *Z*-(13,14-Epoxy)tetradec-11-en-1-ol acetate | | 20.30 | | 1000131-33-2 | | **----** | | **----** | | | 0.06 | |
| **Sum** |  |  | |  | | **8.46** | | **0.47** | | | **1.04** | |  |
| **Phenol** | | | | | | | | | | | | |  |
| **1** | Maltol | 22.52 | | 118-71-8 | | **----** | | | | **----** | 1.95 | |  |
| **2** | Phenol, 5-ethenyl-2-methoxy- | 25.14 | | 621-58-9 | | **----** | | | | **----** | 6.95 | |  |
| **Sum** |  |  | |  | | ---- | | | | ---- | **8.90** | |  |
| **Pyrazine** | | | | | | | | | | | | | |
| **1** | Pyrazine, methyl- | 9.87 | | 109-08-0 | | **----** | | | | **----** | 3.27 | |  |
| **2** | Pyrazine, 2-ethyl-5-methyl- | 18.44 | | 13360-64-0 | | **----** | | | | **----** | 5.99 | |  |
| **3** | Pyrazine, trimethyl- | 18.77 | | 14667-55-1 | | **----** | | | | **----** | 2.57 | |  |
| **Sum** |  |  | |  | | ---- | | | | ---- | **11.82** | |  |
| **Pyridine** | | | | | | | | | | | | | |
| **1** | 4-Pyridinamine, N,N-dimethyl- | 18.10 | | 1122-58-3 | | **----** | | | | **----** | 0.71 | | |
| **2** | 4(H)-Pyridine, N-acetyl- | 20.68 | | 67402-83-9 | | **----** | | | | **----** | 0.18 | | |
| **3** | Ethanone, 1-(2-pyridinyl)- | 20.43 | | 1122-62-9 | | **----** | | | | **----** | 0.30 | | |
| **Sum** |  |  | |  | | ---- | | | | ---- | **1.19** | | |
| **Furan derivative** | | | | | | | | | | | | | |
| **1** | Furan, 2-pentyl- | 15.97 | | 3777-69-3 | | **----** | | | | 3.24 | **----** | | |
| **2** | Benzofuran, 2,3-dihydro- | 24.76 | | 496-16-2 | | **----** | | | | 14.98 | 8.16 | | |
| **Sum** |  |  | |  | | ---- | | | | **18.23** | **8.16** | | |
| **Another nitrogen-containing compound** | | | | | | | | | | | | | |
| **1** | Oxime-, methoxy-phenyl-_ | 16.52 | | 1000222-86-6 | | 0.81 | | | | 3.19 | **----** | | |
| **2** | Naphtho[2,3-b]furan- 2-one, 3- [[(benzo[1,3]dioxol- 5-ylmethyl)amino]methyl]-8a-methyl-5-methylene-decahydro- | 25.16 | | 1000310-95-6 | | **----** | | | | 3.83 | **----** | | |
| **3** | 2-Myristynoyl pantetheine | 2.05 | | 1000111-63-6 | | **----** | | | | **----** | 0.06 | | |
| **4** | Cyclobarbital | 6.35 | | 52-31-3 | | **----** | | | | **----** | 2.58 | | |
| **5** | 4,5-Dimethyl-ortho-phenylenediamine | 21.12 | | 3171-45-7 | | **----** | | | | **----** | 1.82 | | |
| **6** | 4-Pyridinamine,N,N,2,6-tetramethyl- | 22.20 | | 129384-12-9 | | **----** | | | | **----** | 2.33 | | |
| **7** | 5H-5-Methyl-6,7-dihydrocyclopentapyrazine | 22.44 | | 23747-48-0 | | **----** | | | | **----** | 0.18 | | |
| **8** | 1-(1-Phenyl-2-phenylsulfonylaminoethyl)aziridine | 23.02 | | 1000216-13-2 | | **----** | | | | **----** | 0.20 | | |
| **9** | Pilocarpine | 21.46 | | 92-13-7 | | **----** | | | | **----** | 0.14 | | |
| **10** | Imidazole, 2-amino-5-[(2-carboxy)vinyl]- | 2.45 | | 1000116-74-7 | | **----** | | | | **----** | 0.64 | | |
| **11** | 2-Methyl-5,6,7,8-tetrahydroquinoxaline | 22.81 | | 38917-65-6 | | **----** | | | | **----** | 0.18 | | |
| **12** | 5-Thiazoleethanol, 4-methyl- | 25.01 | | 137-00-8 | | **----** | | | | **----** | 4.35 | | |
| **13** | N-[2-[[2-Pyridylmethyl]amino]ethyl]aziridine | 22.89 | | 1000255-62-9 | | **----** | | | | **----** | 0.07 | | |
| **Sum** |  |  | |  | | **0.81** | | | | **7.02** | **12.57** | | |
| **Others** | | | | | | | | | | | | | |
| **1** | Octadecane, 1-(ethenyloxy)- | 19.43 | | 930-02-9 | | 4.43 | | | | **----** | **----** | | |
| **Sum** |  |  | |  | | **4.43** | | | | ---- | ---- | | |

**^†^** ----: not detectable.

**^‡^** The bold part represents the kind of the compound and the total relative content of each class.
